# A customized large single-piece bifrontal implant for post-craniectomy defect reconstruction: A case study

**DOI:** 10.14440/jbm.0019

**Published:** 2025-08-12

**Authors:** Omid Ghaderzadeh, Ehsan Amirbeyk, Seyed Roholah Ghodsi, Zahra Namazi, Lobat Tayebi

**Affiliations:** 1 Department of Research and Development, DanaWell Co., Tehran 158365311, Iran; 2 Department of Neurosurgery, Sabzevar University of Medical Science, Sabzevar, Razavi Khorasan 158365311, Iran; 3 Department of Dental Biomaterials, School of Dentistry, Shahid Beheshti University of Medical Sciences, Tehran 158365311, Iran; 4 Department of Electrical and Computer Engineering, Institute for Engineering in Medicine, Health and Human Performance, Batten College of Engineering and Technology, Old Dominion University, Norfolk, Virginia 23529, United States of America

**Keywords:** Decompressive craniectomy, Large skull defect, Customization, Cranioplasty, Polymethyl methacrylate

## Abstract

**Background:**

Large bifrontal defects pose unique reconstruction challenges due to their complex curvature and mechanical requirements. This case demonstrated how computer-aided design/manufacturing (CAD/CAM) enabled precise single-piece polymethyl methacrylate (PMMA) implant fabrication, thereby overcoming traditional limitations.

**Case presentation:**

A 25-year-old male who had undergone bifrontal decompressive craniectomy suffered a severe traumatic brain injury. The autologous bone flap had been temporarily stored in a subcutaneous fat area of the abdomen for 3 months to preserve its viability. A secondary cranioplasty was then performed using titanium miniplates and self-tapping screws for final fixation. After 2 years, the patient developed empyema and a brain abscess; the infected bone flap was removed. A skull computed tomography (CT) scan was conducted, and a prosthesis was created from PMMA by employing CAD. In the sagittal plane, the defect extended from the frontal bone and surpassed the coronal suture, while in the coronal plane, it reached the temporal region on both sides. The prosthesis was fabricated through rapid prototyping based on CT scan images. Surgery was performed using a patient-specific prosthesis that adequately covered the defect area. Facial aesthetics were restored, and no complications occurred. The patient was followed clinically and radiologically for 1 year, during which no postoperative complications or signs of implant-related issues were observed.

**Conclusion:**

This CAD/CAM single-piece PMMA implant successfully restored large bifrontal defects, suggesting that it may find broader applications in complex cranioplasties and could achieve improved outcomes.

## 1. Background

Traumatic brain injury (TBI) refers to sudden damage to the brain resulting from external mechanical forces such as impact, penetration, or rapid head movement, and is distinct from congenital or degenerative neurological conditions.^1^ As reported in a study by Waltzman *et al.*,^2^ TBIs are a leading cause of mortality and morbidity in the United States.

Although the Glasgow Coma Scale, which rates TBI as mild (13–15), moderate (9–12), or severe (≤8), remains the primary instrument for classifying the severity of TBI, the classification can be affected by confounders such as medication use, alcohol consumption, and medical interventions, including tracheal intubation. Consequently, the duration of post-traumatic amnesia serves as a highly significant predictive indicator of TBI severity and outcomes.^3^ The outcome of TBI is a multidimensional construct influenced by the patient’s social support, ecological, biological, and psychological background.^3^ Intracranial pressure resulting from TBI can lead to mass effects due to intracranial hematomas, diffuse brain swelling, contusions, or hydrocephalus. Multiple studies have focused on the management of intracranial pressure.^4^,^5^ There are several standard medical treatments for reducing intracranial pressure before considering decompressive craniectomy (DC). Due to unfavorable outcomes, DC is considered a last resort for intracranial pressure management.^6^ Cranioplasty not only preserves the normal appearance and physical protection of the brain but can also attain neurological improvement.^7^

Researchers have found that 10% of patients who undergo DC require additional medical and/or neurosurgical interventions (Table 1).^8^ Despite these risks, DC is often unavoidable and remains the only life-saving option in certain cases. After craniectomy, bone flaps are preserved by deep-freezing or by placement in the patients’ abdominal wall. In most cases, bone flaps are unusable, necessitating the use of patient-specific implants or titanium mesh.^9^ Serious complications, such as meningitis, air embolism, and death, are rare, although high complication rates have been reported following cranioplasty.^10^ For large cranial defects, computer-aided manufacturing offers superior outcomes compared to manual mesh shaping, including improved aesthetic results, reduced operative time, and fewer postoperative complications.^11^ However, large metallic implants may cause thermal sensitivity and skin discoloration.^10^ In addition, cranioplasty with metallic implants limits the procedure to the predefined geometry of the implant, as intraoperative drilling or milling is not feasible.

**Table 1:** Overview of complications associated with decompressive craniectomy^8^

Stage	Decompressive craniectomy
Early	• Hemorrhage (hematoma expansion)
	• External cerebral herniation
	• Wound complications
	• CSF leak/fistulae
	• Postoperative infection
	• Seizures/epilepsy
Late or delayed	• Subdural hygroma
	• Hydrocephalus
	• Syndrome of the trephined

Abbreviation: CSF: Cerebrospinal fluid

Polymethyl methacrylate (PMMA) is widely used in orthopedic and neurosurgical fields due to its proven biocompatibility and favorable mechanical properties. Major advantages include chemical inertness, acceptable thermal conductivity, radiolucency, and cost-effectiveness. Furthermore, PMMA surpasses alternative bone substitutes in clinical utility owing to its intraoperative malleability and ease of application.^12^,^13^ Oliver *et al*.^12^ studied 1,459 cases of cranial PMMA prosthesis cranioplasty in 2019, reporting an infection rate of 7.95% (*p*=0.0266). Implants used in the frontal or temporo-basal areas must fit precisely, conform completely to skull anatomy, and meet cosmetic expectations.

Conventional intraoperatively fabricated implants expose surrounding tissues to the heat generated by the exothermic polymerization of methyl methacrylate during the curing process. In contrast, rapid prototyping-manufactured implants eliminate this thermal risk through preoperative fabrication, while maintaining precise anatomical conformity. Various fixation devices, including mini plates, mesh plates, and silk threads, can be utilized to secure PMMA implants. The drillability of customized PMMA implants offers significant intraoperative advantages, particularly when managing anatomical uncertainties or unexpected surgical findings. This adaptable feature allows for real-time modifications and secure fixation during the procedure. The flexibility in positioning drill sites allows the surgeon to use screws, plates, or silk threads in any desired area.

## 2. Case presentation

### 2.1. Patient and surgery details

**Figure 1:**
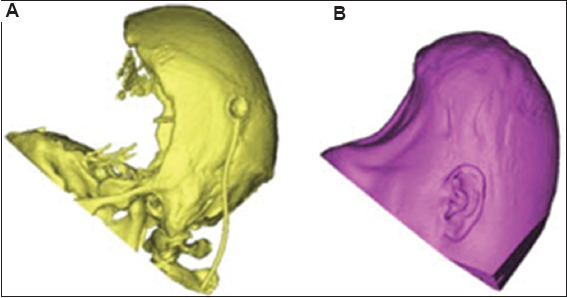
Patient’s (A) hard tissue and (B) soft tissue after craniectomy

**Figure 2:**
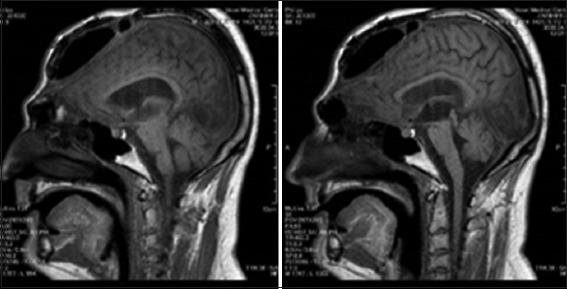
Magnetic resonance images obtained after bone flap fixation

The subject presented in this study was a 25-year-old male with severe TBI. A DC was performed to reduce intracranial pressure (Figure 1). The skull bone flap was placed in the subcutaneous fat area of the abdomen for 3 months. In addition, the dura mater was enlarged by grafting surrounding skull tissues to provide more space for possible brain edema. After 3 months, surgery was performed to restore the skull bone flap (Figure 2). Unfortunately, the patient developed hydrocephalus, and shunting surgery was subsequently performed.

Two years later, the patient developed empyema and a brain abscess. The bone flaps on both frontal sides of the skull were removed, washed, treated, and the area was closed.

**Figure 3:**
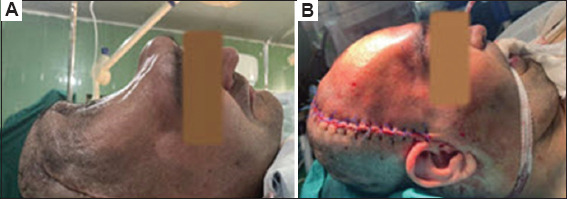
Patient appearance (A) before and (B) after cranioplasty

With the bone flaps removed, the brain was left unprotected, and as shown in Figure 3, the scalp was in direct contact with the brain. In this situation, brain dynamics might be disrupted, potentially causing a syndrome of the trephined. Due to the large defect size and the impracticality of fabricating a prosthesis intraoperatively or forming titanium meshes, an alternative method was necessary.

### 2.2. Customized implant fabrication

Given the patient’s condition and constraints, the only feasible option was to fabricate the cranial prosthesis preoperatively using computed tomography (CT) imaging and rapid prototyping techniques. High-resolution CT data with a slice thickness of <1 mm were used to generate a 3D reconstruction of the cranial defect. For 3D modeling and anatomical segmentation, Materialise Mimics^®^ software (Materialise NV, Belgium) was used. The resulting digital model served as the basis for designing and fabricating a patient-specific PMMA implant.

**Figure 4:**
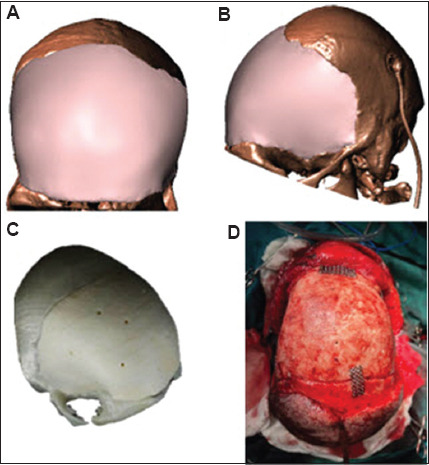
Computer-aided design model of the customized implant: (A) front view, (B) side view, (C) pre-surgical model, and (D) implantation

A common design approach for cranial prostheses is mirroring the size and contour of the opposite side. However, this method was not applicable in this case due to the bilateral extent of the defect. To design the patient-specific bifrontal prosthesis, we utilized skull CT scans from 10 anatomically normal controls (with no frontal or parietal defects) combined with advanced scaling algorithms. This approach enabled the creation of a single-piece implant (defect area: 250 cm^2^) with precise topographic alignment to the defect margins (Figure 4). Despite the technical challenges inherent in large-scale cranial reconstruction, the prosthesis was successfully fabricated using additive manufacturing protocols that maintained complete continuity with adjacent cranial curvature. The defect size was obtained by measuring the prosthesis’s total surface area in CATIA (where the Measure Inertia tool was chosen; Dassault Systèmes, France).

### 2.3. Final cranioplasty

Given the extensive defect size and unitary implant construction, a patient-specific anatomical model was preoperatively fabricated to (i) verify precise congruence between the prosthesis and the defect margins and (ii) enable surgical rehearsal, allowing the team to optimize positioning, fixation points, and installation techniques before the actual procedure. Using this model, the surgeon evaluated the defect and prosthesis placement on the skull before surgery. The surgery was then performed using a customized PMMA implant. Implant fixation was accomplished using titanium screws and mesh.

### 2.4. Aesthetic result evaluation

To assess the patient’s satisfaction with the aesthetic outcome, the questionnaire in Table 2, proposed by Fischer *et al.*,^14^ was administered and completed by the patient’s parents. The questionnaire was used at 3 and 12 months post-surgery.

**Table 2:** Aesthetic outcome assessment criteria based on Fischer *et al.*^14^

Item no.	Question	Answer
1.	Please estimate the size of your implant:	Small/medium/large
2.	Choose one of the statements that best describes your satisfaction with the aesthetic result of the cranioplasty:	a. I do not accept the aesthetic result after cranioplasty and would like to improve the appearance with another surgical intervention b. I am not satisfied with the aesthetic result c. I am satisfied with the aesthetic result d. I am very satisfied with the aesthetic result and think that cranioplasty does not impair my appearance at all
3.	If you are dissatisfied, please indicate the reason for your dissatisfaction:	For example, dents, bulges, scars, or irregular bone edges
4.	Did your level of satisfaction change over time after cranioplasty?	“Free-form”
5.	Did you have any medical complications after the cranioplasty?	“Free-form”

The production and use of a large single-piece bifrontal implant reduced the need for extra screws and plates during surgery, which is particularly important for the patient’s appearance, especially considering that the implant covered both the frontal and bilateral temporal regions.

**Figure 5:**
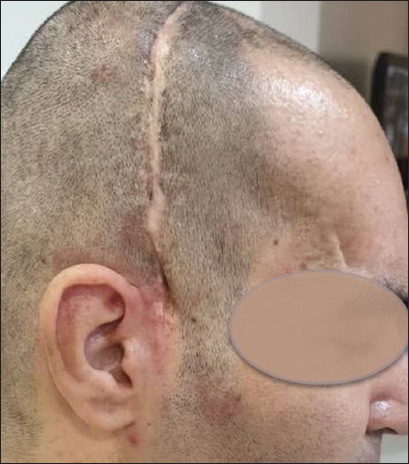
Patient’s condition 1 year after cranioplasty with a customized polymethyl-methacrylate prosthesis.

As mentioned above, after 3 months of implant placement, the patient’s parent was contacted and asked questions outlined in Table 2. During the 1-year follow-up period, the results have been very successful (including satisfactory aesthetic outcome and complete absence of infection) (Figure 5). In addition, medical examinations showed no signs of infection in the patient. According to the patient’s family, although brain function progress was halted for the first few weeks, improvement resumed approximately 1 month after the cranioplasty.

## 3. Discussion

Although DC significantly increases the survival rate in patients with severe TBI, complications, including severe disabilities, are often inevitable. Cranioplasty not only restores the physical barrier and aesthetics but also contributes to neurological improvement. Additive manufacturing enables the rapid fabrication of patient-specific PMMA implants that achieve precise anatomical conformity and demonstrate clinical practicality, even for large-scale cranial reconstructions.

One of the most notable innovations in this case is the successful design and implantation of a large, single-piece bifrontal cranial prosthesis made entirely of PMMA. Unlike multi-segmented implants, which may present interlocking challenges and aesthetic mismatches during surgery, the single-piece construct offers precise anatomical conformity and preserves the natural curvature of the cranial vault. This is particularly critical in bifrontal reconstructions, where cosmetic symmetry is essential. Moreover, multi-piece solutions may fail to align properly during fixation, resulting in gaps or uneven surfaces that compromise both aesthetics and function. Our approach overcame these limitations by employing a preoperative digital workflow and rapid prototyping techniques, resulting in a unified implant that ensured secure fixation and exerted a positive impact on the patient’s facial appearance.

One of the major challenges in reconstructing large cranial defects is the risk of prosthesis exposure, especially when metallic implants or mesh are used. Custom PMMA prostheses effectively address this issue due to their biocompatibility and superior adaptability. Moreover, the single-piece design in this case ensured continuous surface integrity and avoided alignment problems commonly seen with multi-piece implants, which can lead to gaps or uneven surfaces that negatively impact cosmetic outcomes.

PMMA and polyether ether ketone (PEEK) implants are both widely accepted as suitable materials for patient-specific cranial reconstruction. However, compared to traditional metallic options, both PMMA and PEEK offer significant advantages. Metallic implants, while strong, may cause thermal sensitivity, skin discoloration, and limited intraoperative adaptability. In contrast, PMMA allows for real-time cutting and drilling during surgery, enhancing intraoperative flexibility and customization. The implant used in this case benefited from these properties, offering a secure fit with minimal hardware and contributing to successful functional and aesthetic outcomes.

## 4. Conclusion

This study demonstrated that a customized single-piece PMMA implant successfully addressed major cranioplasty challenges for large bifrontal defects, achieving precise anatomical fit with reduced fixation hardware while preserving aesthetic contours. The implant’s intraoperative adaptability and biocompatibility overcame limitations of multi-part/metal prostheses, highlighting PMMA’s utility in complex reconstructions.

The single-piece design represents a paradigm shift by eliminating segment alignment issues and optimizing cranial symmetry. Future work should explore screwless fixation techniques, growth-adjustable pediatric implants, and temporal hollowing correction. Standardizing this approach could transform management of extensive cranial defects, particularly in trauma and pediatric cases requiring dynamic solutions.

## Data Availability

The datasets used and analyzed in this study are available from the corresponding author upon reasonable request. Data sharing complies with institutional ethical guidelines.
